# Attitudes Toward Sexual Behaviors in Long-Term Care: Development of an Assessment Tool

**DOI:** 10.1093/geroni/igaa057.2031

**Published:** 2020-12-16

**Authors:** Rachael Spalding, Emma Katz, Barry Edelstein

**Affiliations:** 1 West Virginia University, Morgantown, West Virginia, United States; 2 WVU, Morgantown, West Virginia, United States

## Abstract

Most older adults living in long-term care settings (LTCs) indicate that expressing their sexuality is important to them (Doll, 2013). Little is known about the general public’s attitudes towards sexual behaviors in LTCs. Attitudes of LTC residents’ family members are particularly important, as family members are most likely to visit residents and to care about their quality of life. Family members’ attitudes could in turn inform facility policies and management. We will present preliminary data from a series of qualitative interviews with community-dwelling adults regarding their attitudes. We will discuss how these data are being used to inform current work on a measure of attitudes toward sexual behavior in LTCs.

